# Enhancing growing rabbit heat stress resilience through dietary supplementation with natural antioxidants

**DOI:** 10.1186/s12917-024-04466-1

**Published:** 2025-01-18

**Authors:** Ibrahim Talat El-Ratel, Aml Mekawy, Sara H.M. Hassab, Sameh Abdelnour

**Affiliations:** 1 Department of Animal, Poultry and Fish Production, Faculty of Agriculture, Damietta University, Damietta, 34517 Egypt; 2 Department of Animal Production, Faculty of Agriculture, Zagazig University, Zagazig, 44511 Egypt

**Keywords:** Vitamins, Heat stress, Antioxidants, Redox status, Immunity, Growing rabbits

## Abstract

Animal husbandry development is influenced by various factors, with heat stress (HS) being a significant factor. The aim of this experiment was to explore the potential of natural antioxidants such as vitamin C (VITC), vitamin E (VITE), lycopene (LYC), and allicin (AL) in enhancing growth, immune function and maintaining the redox status of fattening rabbits under HS. Male weaning rabbits (*n* = 150, 5 weeks of age) were randomly assigned to 5 groups. The rabbits were fed a basal diet (control group) or supplemented with 40 mg of vitamin E (VE40), 5 mg of vitamin C (VC5), 150 mg of lycopene (LYC150), or 150 mg of allicin (AL150) per kg of diet, respectively, under summer Egyptian conditions. The overall temperature humidity index (THI) value was 29.76, indicating severe HS during the experimental period. The findings indicated that all dietary supplemented groups showed significant improvements in live body weight at 8 weeks (*P* < 0.0001) and 11 weeks (*P* < 0.05) of age compared to the control group. The feed conversion ratio (FCR) was improved with all additives (*P* < 0.05), while feed intake and carcass traits were not affected by the treatments (*P* > 0.05). The AL group had the highest dressing percentage compared to the other groups (*P* < 0.05). Feeding stressed rabbits with antioxidant supplements resulted in a higher hemoglobin concentration compared to the control group (*P* < 0.05). Aspartate transaminase (AST), triglycerides, and creatinine levels were decreased with all additives as compared to the control group (*P* < 0.05). Total protein and albumin were significantly higher in AL group than in other groups (*P* < 0.05). The serum Immunoglobulin G (IgG) was significantly increased, while tumor necrosis factor alpha (TNF-α), interleukin-4 (IL-4), and interferon gamma (IFN-γ) were decreased by all feed additives (*P* < 0.05). Immunoglobulins (IgA and IgM) did not differ among all experimental groups (*P* > 0.05). Serum total antioxidant capacity (TAC) and glutathione (GSH) levels were higher in all supplement groups compared to the HS group (*P* < 0.05). All dietary supplements significantly reduced malondialdehyde (MDA) levels in liver tissues and blood serum compared to the control group (*P* < 0.05). Collectively, allicin emerged as a potent shield against heat stress, bettering lycopene and vitamins E and C in safeguarding the well-being of growing rabbits.

## Introduction

The adverse effects of global climate change pose significant risks to food security, particularly within the livestock sector. Heat stress (HS) is a major environmental stressor that leads to substantial economic losses in the livestock industry [1]. HS results from an organism’s inability to dissipate heat effectively, leading to a potentially lethal increase in core body temperature [2]. The global rabbit industry is expanding rapidly to meet the growing demand for meat, functional foods, and pharmaceutical products [2]. Rabbit meat is favored because it contains high protein, vitamin, and mineral contents as well as low saturated fatty acid content [2]. Rabbits are suffering from HS during hot conditions in summer due to lacking sweat glands [3]. Elevated temperatures can reduce growth and feed utilization [4, 5] by damaging intestinal barriers [6], leading to decreased nutrient absorption and metabolism [7, 8]. HS also alters blood hematology, causing anemia and disruptions in the physiological balance of the cellular system, which can result in immune dysfunction [9, 10]. Increased oxidative stress (OS) due to HS conditions can trigger lipid peroxidation, promote inflammation [3, 11], and hinder growth, productivity, and overall health. To enhance the health of growing rabbits in hot climates, different approaches have been employed, such as genetic enhancements, nutritional interventions, and management practices.

Many feed supplements can help decrease the impact of HS in rabbits [12] and enhance their performance. Conversely, antibiotics and chemical substances may have negative side impacts, such as promoting antibiotic resistance in pathogenic bacteria [13]. Additionally, some antibiotic can be harmful pollutants and pose risks to consumer health [14]. Therefore, using natural substances as feed supplements to boost growth, health, and reproductive performance is a more sustainable and safer approach [12].

Vitamin C (ascorbic acid, VC), a water-soluble vitamin, plays an important role in preventing cells from OS and modulating the immune response [9, 15]. It also serves as a co-factor in various enzymatic reactions. VC (250 mg/kg diet in growing rabbits, while 30 mg/kg b.w of bucks ) has been shown to improve growth markers, and blood health in rabbits exposed to HS conditions and normal temperatures [9, 16]. Vitamin E (α-tocopherol, VE) is essential for various important functions in the body, including cellular signaling, growth, reproduction (150 mg/kg diet) antioxidant protection, immunity, and disease resistance [17]. Several reports have confirmed that adding VE to the diet can enhance the growth indices [5, 9] and reproductive aspects [17] of rabbits in high-stress conditions.

Lycopene (LYC) is a predominant carotenoid in tomatoes and tomato products. It has been found to increase the levels of antioxidant enzymes through the stimulation of antioxidant response elements in DNA [18, 19]. Supplementation of lycopene (50 mg/kg, b.w.) in heat-stressed bucks improved blood hematology, antioxidant capacity and semen quality [16]. LYC has been shown to alleviate testicular damage induced by lipopolysaccharide via integrating lipid metabolism and reducing inflammation response [18]. LYC can also improve liver health during HS conditions in rabbits [20], but its significant roles in other blood metabolites, inflammation and oxidative stress in growing rabbits remain unclear.

Allicin (AL) is the main organosulfur compound found in garlic [21]. It has the ability to combat bacteria, fungi, inflammation [22], and oxidation [21]. Additionally, AL has properties that inhibit bacteria growth, decrease OS [8, 23], improve energy efficiency, viability, and modulate the immunity of growing rabbits [8, 20, 24, 25]. Limited studies have investigated the use of LYC or AL in rabbits’ diets with a narrow focus on specific parameters [16, 19, 20, 25]. Despite a thorough review of the literature, no comparative studies were found to evaluate the effectiveness of vitamin E, vitamin C, lycopene, or allicin in mitigating the adverse effects of heat stress in growing rabbits. Based on previous research on the biological effects of AL or LYC, we hypothesize that natural antioxidants such as AL and LYC, when used as dietary supplements will enhance growth efficiency, feed utilization, blood health, immune function, and antioxidant capacity in thermally stressed growing rabbits compared to vitamins E or C.

## Materials and methods

### Ethical statement and sources of feed additives

The experiment and all animal procedures were checked and authorized by the Animal Care and Use Committee of the Laboratory Animal Center of Zagazig University, Egypt (Ethics Approval Number: ZU-IACUC/2/F/367/2022). All methods in this study were conducted in compliance with the relevant guidelines and regulations of ZU-IACUC and the Animal Research Reporting of In Vivo Experiments (ARRIVE) guidelines. Both vitamins E and C were purchased from MISR company for Food Additives, Badr City, Egypt. Allician was provided by Cangzhou Biocon Biotechnology Co., Ltd company, China, while the lycopene was acquired from Pure Life for Agricultural Services (Giza, Egypt).

### Animal and experimental groups

The experiment was conducted at Damietta University, Faculty of Agriculture, Damietta, Egypt, for eight consecutive weeks during the hot period (July and August). The rabbits were provided by the rabbit farm unit, Faculty of Agriculture, Damietta University. In this experiment, 150 weaned male APRI rabbits (5 weeks old, average body weight 671.04 ± 6.45 g) were used. The animals were randomly divided into five groups with 30 animals in each group. The first group (control) was fed the basal diet. The 2nd, 3rd, 4th, and 5th groups of rabbits were fed basal diet supplemented with 40 mg vitamin E (VE40), 5 mg vitamin C (VC5), 150 mg lycopene (LYC150), and 150 mg allicin (AL150) per kg of diet, respectively. The feed supplements were integrated into the diet during the manufacturing process. During the experimental period from 5 to 11 weeks of age, rabbits were individually housed in galvanized wire battery cages (50 × 50 × 40 cm) located in a naturally ventilated building. All groups were raised under the same management and hygiene conditions. Rabbits were fed a basal pelleted diet *ad libitum* and had access to fresh tap water. The ingredients and chemical composition of the basal diet, designed to meet the nutritional requirements of growing rabbits according to [26], are shown in Table 1.

**Table 1:** Constituents and chemical assessment of the basal diet fed to the fattening rabbits

Ingredient	g/1000 g diet	Analyzed composition (%, on DM basis)	
Berseem hay	300.5	Organic matter	91.42
Wheat brain	215.0	Crude protein	17.04
Soybean meal	175.0	Crude fiber	12.37
Barley grain	246.0	Ether extract	2.23
Limestone	9.5	Nitrogen free extract	59.46
Molasses	30.0	Ash	8.58
Di-calcium phosphate	16.0		
DL-Methionine	1.5		
Sodium chloride	3.0		
Vitamins & Mineral Premix (1)	3.5		
Total	100		

1) Each 1 kg contains on Vitamin A (150, 000 UI), Vitamin B12 (0.1 mg), Vitamin B1(10 mg), Vitamin K3 (21 mg), Vitamin B6 (15 mg), Folic acid (10 mg) VitaminB2 (40 mg), Pantothenic acid (100 mg) Niacin (200 mg), Vitamin E (100 mg), Biotin (0.5 mg), and Choline chloride (5000 mg). Each 1 kg contains zinc (600 mg), manganese (800 mg), copper (40 m g), iron (300 mg), iodine (500 mg), selenium (100 mg), and cobalt (100 mg)

### Climatological factors

In the rabbitry, the relative humidity (RH, %) and ambient air temperature were assessed daily using an automatic Thermo-hygrometer (Dostmann GmbH and Co. KG, Wertheim, Germany). The THI (temperature-humidity index) was then calculated using the following equation: THI = dp−[(0.31 − 0.31(RH100))×(dp − 14.4)] [4], where dp is the dry bulb temperature in Celsius (°C). The THI evaluations were categorized as follows: absence of HS < 27.8; moderate HS, 27.8 to 28.9; severe HS 29.0 to 30.0; and very severe HS > 30.0.

### Growth, carcass traits and chemical composition

The growth variables including live body weight (LBW), body weight gain (BWG), feed intake, and feed conversion ratio (FCR, g feed/g gain) were determined at age intervals of 5–8, 8–11, and 5–11 weeks of age. At 11 weeks of age, 6 rabbits from each group were selected, fasted for 12 h, weighed, and sacrificed following the Islamic method. After slaughtering and complete bleeding, the carcass was portioned into fore, loin, hind, and toe, then weighed and calculated as a percentage of the pre-slaughter weight. The dressing percentage (hot-dressed carcass weight/pre-slaughter weight x100) was calculated [27]. To assess crude protein (CP), fat and ash, 100 g of meat were dried and used for calculating the chemical composition. The CP was determined by the Kjeldahl method using a Buchi analyzer (Centec Automatika, Prague, Czech Republic), while the ether extract content was measured by the Soxhlet method (Thermo Scientific, Warrington, UK). The dietary samples were incinerated at 550 °C for ash content determination according to [27].

### Blood hematology

According to Islamic guidelines for animal slaughter, anesthesia is not required during the process. During the slaughtering process, two blood samples per animal were collected from five rabbits in each group. The first sample was taken in sterile tubes with anticoagulant (heparin) for evaluating the hematological parameters in whole blood, following the method described in [28] using an automated hematology analyzer (Hospitex Hema Screen 18, Sesto Fiorentino, Italy). The second sample was allowed to clot, then centrifuged for 15 min at 1507 *g* to obtain serum samples which were stored at − 20 °C until biochemical analyses. Hematological parameters such as hemoglobin (Hb, g/dL), red blood cells (RBCs,10^6^/mm^3^), white blood cells (WBCs,10^6^/mm^3^), platelets and packed cell volume (PCV, %) were assessed.

### Blood chemistry and redox status

The concentration of biochemical markers, such as total protein (TP), albumin, triglycerides, total cholesterol, creatinine, and urea as well as the activity of liver enzymes including aspartate (AST) and alanine (ALT) transaminases were assayed in blood serum using a spectrophotometer and commercial kits (Bio-diagnostic Company, Giza, Egypt), following the manufacturer’s instructions. Globulin concentration was determined by the difference between TP and albumin.

The total antioxidant capacity (TAC), activity of antioxidant enzymes like superoxide dismutase (SOD), and glutathione peroxidase (GSH-Px), as well as level of glutathione (GSH) and malondialdehyde (MDA) in blood serum were determined using specific commercial kits according to the manufacture’s procedures (BioMérieux, Marcy-l’Etoile, France).

### TAC and MDA in liver tissue

To assess the TAC and MDA levels in liver tissues, liver specimens (500 mg) were homogenized in chilled phosphate saline buffer. The hepatic tissues (100 mg) were homogenized in 1 mL of lysis buffer using a homogenizer. The samples were then centrifuged at 3000 rpm for 30 min. The liquid portion was used to detect the levels of TAC and MDA following the manufacturers guidelines (BioMérieux, Marcy-l’Etoile, France).

### Blood immunoglobulins and inflammatory biomarkers

The levels of immunoglobulins (IgG and IgA) in the serum of experimental rabbits were measured using a previously described method [29]. Inflammatory responses, including tumor necrosis factor-alpha (TNF-α) [30], interferon-gamma (IFN-γ) [31] and interleukin-4 (IL-4) [32], were also detected in the treated and control rabbit’s serum. The Elisa kits for inflammatory biomarkers were obtained from MyBioSource (San Diego, CA, USA) and used according to the manufacturer’s instructions. Additionally, serum lysosomal activity was evaluated using a 96-well microplate turbidity assay as described by [33].

### Statistical analysis

The data was recorded in Microsoft Office Excel version16. Normality was checked using a Shapiro-Wilk test as described by [34]. Growth, feed utilization, carcass and chemical indices, hematology profile, redox status, immunity factors, and inflammatory reactions were assessed using the MIXED procedure (PROC MIXED; SAS Institute, 2012). The following statistical model was applied to the data:

Yij = µ + T_i_ + e_ij_.

Y_ij_ is the indicated value of the concerned treatment, µ is the indicated mean for the concerned treatment, T_i_ is the treatment effect, and e_ij_ is the error related to individual observation.

Individual rabbits were considered a random factor, and antioxidant supplements were treated as a fixed factor. Multiple comparisons between means were performed using Duncan’s multiple range test [35]. A *P* value is less than 0.05, considered significant.

## Results

### THI indices

The overall THI value was 29.76, indicating severe heat stress during the experimental period (Table 2).

**Table 2:** The average values of AT, RH, and THI during the research

Item	July	August	Overall
**AT (°C)**	32.14 ± 0.19	31.11 ± 0.25	31.62 ± 0.24
**RH (%)**	63.83 ± 1.11	66.38 ± 2.18	65.10 ± 0.85
**THI value**	30.15 ± 0.13	29.36 ± 0.19	29.76 ± 0.20

Relative humidity (RH), ambient temperature (AT), temperature–humidity index (THI)

### Performance parameters

All dietary supplemented groups showed significant improvements in LBW at 8 (*P* < 0.0001) and 11 (*P* < 0.05) weeks of age (Table 3). There were no notable differences among all treated groups in terms of LBW (*P* > 0.05). Although the ADG was lower in the HS group, there were no significant variations observed among all groups between 8 and 11 weeks of age (*P* = 0.91. Overall, the growing rabbits that were fed diets enriched with VE, VC, LYC150, and AL150 exhibited greater ADG compared to the HS group. However, there were no substantial variations in FI observed among all experimental groups (*P* > 0.05) during the specified periods. Only at 5–8 weeks of age, the AL150 group showed a higher FCR compared to the HS group (*P* < 0.05), while the other groups had similar FCR values to the HS group (*P* > 0.05).

**Table 3:** Impacts of dietary different sources of antioxidants supplementation on growth parameters and feed efficiency of fattening rabbits exposed to heat stress conditions

Variables	Experimental groups^1^					SEM	*P*-value
	CON	VE40	VC5	LYC150	AL150		
**Live body weight (LBW, g)**							
5 weeks	672.36	671.04	672.56	670.52	673.52	6.45	0.991
8 weeks	1255.32^b^	1308.84^a^	1316.88^a^	1321.56 ^a^	1323.08^a^	9.84	< 0.001
11 weeks	1905.56 ^b^	1954.84 ^a^	1950.00 ^a^	1963.16^a^	1968.44^a^	14.82	0.027
**Average daily gain (ADG**,** g/day)**							
5–8 weeks	27.75 ^b^	30.37 ^a^	30.68^a^	31.00^a^	30.93^a^	0.39	< 0.0001
8–11 weeks	30.96	30.76	30.14	30.55	30.7316	0.61	0.905
5–11 weeks	29.36^b^	30.56^a^	30.42^a^	30.77^a^	30.83^a^	0.34	0.020
**Feed intake (FI**,** g /day)**							
5–8 weeks	96.040	95.56	97.64	96.72	95.32	3.19	0.986
8–11 weeks	138.08	135.48	136.00	136.12	135.96	2.99	0.977
5–11 weeks	125.32	125.88	127.40	127.96	126.84	2.95	0.951
**FCR (g feed/g gain)**							
5–8 weeks	3.47^a^	3.16^ab^	3.18^ab^	3.13^ab^	3.09 ^b^	0.11	0.139
8–11 weeks	4.46	4.41	17.57	4.47	4.43	5.93	0.422
5–11 weeks	4.27	4.12	4.26	4.16	4.12	0.11	0.762

^ac^ Means (*n* = 30) not sharing a frequent superscript in a row are significantly different (*P* < 0.05). Average daily gain (ADG), live body weight (LBW), feed intake (FI), and feed conversion ratio (FCR). ^1^ Growing rabbit fed basal diet without feed additive (CON group and serve as HS group) or fed diets with 40 mg of vitamin E (VE40), 5 mg of vitamin c (VC5), 150 mg of lycopene (LYC150) or 150 mg/kg diet of allicin (AL150) for 8 weeks during natural heat stress

### Carcass criteria

The impacts of various sources of antioxidant supplements on the carcass traits of growing rabbits exposed to HS conditions are clarified in Table 4. There were no significant effects of antioxidant supplementation on the fore, loin, hind, teo and the chemical composition of meat such as CP, fat, and ash (*P* > 0.05), except for dressing percentage (*P* < 0.05). AL150 group resulted in greater dressing percentages than that of the HS group (*P* < 0.05), while the other treated groups had similar data for dressing percentages compared to control one (*P* > 0.05).

**Table 4:** Effects of dietary different sources of antioxidants supplementation on carcass traits and chemical composition of growing rabbits exposed to heat stress conditions

Variables	Experimental groups^1^					SEM	*P*-value
	CON	VE40	VC5	LYC150	AL150		
Pre-slaughter (g)	1927.20	1980.00	1985.00	1985.20	1962.20	22.08	0.32
Dressing (%)	57.03^b^	57.61^b^	57.81^b^	59.17^ab^	60.88^a^	0.86	0.03
Fore (%)	28.69	26.85	26.53	26.40	25.91	0.86	0.22
Loin (%)	21.95	21.83	21.70	21.35	21.01	0.73	0.88
Hind (%)	31.79	32.02	32.12	32.15	32.08	0.41	0.97
Toe (%)	17.56	19.29	19.65	20.09	21.00	1.45	0.56
**Chemical composition (%)**							
Crude protein (%)	18.49	18.44	18.41	18.47	18.45	0.57	1.00
Fat (%)	3.60	3.52	3.51	3.59	3.52	0.11	0.96
Ash (%)	1.95	1.97	1.96	1.96	1.97	0.06	0.99

^ac^ Means not sharing a common superscript in a row are significantly different (*P* < 0.05). ^1^ Growing rabbit fed basal diet without feed additive (CON group and serve as HS group) or fed diets with 40 mg of vitamin E (VE40), 5 mg of vitamin c (VC5), 150 mg of lycopene (LYC150) or 150 mg/kg diet of allicin (AL150) for 8 weeks during natural heat stress

### Blood haematology and chemistry

The effects of dietary supplementation with different sources of antioxidants on haemato-biochemical parameters of stressed growing rabbits are reported in Table 5. No significant impact of dietary treatments was observed on hematological parameters such as RBCs, WBCs, platelets, and PCV (*P* > 0.005). However, all supplemented stressed groups showed a higher concentration of Hb compared to the untreated group (*P* < 0.05). RBCs and platelets tended to be higher in all feed additive groups, although there were no significant differences compared to the control group (*P* > 0.05). The levels of ALT, globulin, and AL/GL ratio were not affected by the dietary feed additives during HS conditions (*P* > 0.05). The stressed rabbits fed diets with various feed additives exhibited significantly lower levels of AST, triglycerides, and creatinine compared to the untreated group (*P* < 0.05). Specifically, the dietary supplementation with 150 mg of AL/kg diet resulted in a significantly higher total protein level than the other groups (*P* < 0.05). HS affects rabbits by significantly increasing urea, and total cholesterol, while significantly decreasing albumin. The AL150 group had higher albumin levels compared to the VC5, VE40, and HS groups (*P* < 0.05). The LYC150 and VC5 groups showed similar results for urea and total cholesterol levels (*P* > 0.05).

**Table 5:** Influences of various dietary sources of antioxidants supplementation on haemato-biochemical parameters of fattening rabbits exposed to heat stress conditions

Variables	Experimental groups^1^					SEM	*P*-value
	CON	VE40	VC5	LYC150	AL150		
**Hematological parameters**							
Hb (g/dL)	10.82^b^	12.06^a^	12.16^a^	12.32^a^	12.29^a^	0.318	< 0.001
RBCs (10^6^/mm^3^)	5.24	5.33	5.40	5.50	5.55	0.130	0.504
WBCs (10^3^/mm^3^)	6.55	6.04	6.11	6.12	6.14	0.150	0.166
Platelets (10^3^/mm^3^)	269.10	271.03±	288.23	291.57	295.33	9.620	0.216
PCV (%)	31.72	29.30	30.92	29.79	30.76	1.75	0.874
**Biochemicals parameters**							
Total proteins (TP, g/dL)	6.39^c^	6.64^b^	6.76^b^	6.82^b^	7.13^a^	0.47	< 0.0001
Albumin (AL, g/dL)	3.61^c^	3.81^bc^	3.89^bc^	3.98^ab^	4.27^a^	0.11	0.006
Globulin (GL, g/dL)	2.78	2.83	2.86	2.84	2.86	0.13	0.990
AL/GL ratio	1.31	1.35	1.38	1.43	1.51	0.10	0.699
Triglycerides (mg/dL	82.45^a^	69.10^b^	66.21^b^	63.01^b^	60.09^b^	3.23	< 0.001
Total cholesterol (mg/dL)	198.32^a^	169.11^b^	160.11^bc^	155.76^bc^	150.19^c^	5.47	< 0.0001
Creatinine (mg/dL)	1.13^a^	1.01^ab^	0.92^b^	0.91^b^	0.94^b^	0.06	0.0601
Urea (mg/dL)	39.51^a^	36.83^ab^	33.69^bc^	32.86^bc^	30.47^c^	1.39	0.001
AST (IU)	34.92^a^	30.22^b^	27.13^b^	27.10^b^	26.20^b^	1.43	0.001
ALT(IU)	51.53	46.07	46.24	46.03	44.93	2.56	0.412

^ac^ Means not sharing a common superscript in a row are significantly different (*P* < 0.05). hemoglobin (Hb), red blood cells (RBCs), white blood cells (WBCs), albumin/ globulin ratio (AL/GL), packed cell volume (PCV), aspartate transaminase (AST), alanine transaminase (ALT). ^1^ Growing rabbit fed a basal diet without feed additive (CON group and serve as HS group) or fed diets with 40 mg of vitamin E (VE40), 5 mg of vitamin C (VC5), 150 mg of lycopene (LYC150) or 150 mg/kg diet of allicin (AL150) for 8 weeks during natural heat stress

### Antioxidant capacity in blood and liver tissue

Supplementation with various antioxidants did not have a significant impact on the serum concentration of SOD and GSH-Px compared to the HS group (*P* > 0.05; Table 6). However, TAC and GSH levels in the serum of growing rabbits fed diets enriched with various antioxidants were significantly greater than those in the HS group (*P* < 0.05). Supplementing with 150 mg/kg of AL significantly decreased the MDA levels in the blood serum of growing rabbits compared to the HS group, while no significant effects were noted among all treated groups for MDA (Table 6).

**Table 6:** Influence of dietary different sources of antioxidants supplementation on antioxidants ability in blood and liver tissue of growing rabbits exposed to heat stress conditions

Variables	Experimental groups^1^					SEM	*P*-value
	CON	VE40	VC5	LYC150	AL150		
**Antioxidants capacity in blood serum**							
TAC (ng/mL)	0.37^b^	0.48^a^	0.51^a^	0.53^a^	0.57^a^	0.04	0.009
GSH (mg/dL)	11.89^b^	13.54^a^	13.88^a^	13.99^a^	14.08^a^	0.51	0.036
SOD (U/mL)	0.31	0.35	0.36	0.35	0.39	0.03	0.387
GSH-Px (mg/dL)	5.19	5.38	5.42	5.45	5.44	0.21	0.907
MDA (nmol/mL)	13.01^a^	11.20^ab^	11.01^ab^	11.03^ab^	10.18^b^	0.81	0.013
**Antioxidants capacity in liver tissue**							
TAC (nmol/mL)	1.11^b^	1.32^ab^	1.30^ab^	1.40^ab^	1.54^a^	0.1	0.038
MDA (nmol/mL)	4.23^a^	4.01^ab^	3.87 ^b^	3.84^b^	3.79^b^	0.09	0.013

^ac^ Means not sharing a common superscript in a row are significantly different (*P* < 0.05). Total antioxidant capacity (TAC), malondialdehyde (MDA), superoxide dismutase (SOD), glutathione (GSH), glutathione peroxidase (GSH-Px). ^1^ Growing rabbit fed a basal diet without feed additive (CON group and serve as HS group) or fed diets with 40 mg of vitamin E (VE40), 5 mg of vitamin c (VC5), 150 mg of lycopene (LYC150) or 150 mg/kg diet of allicin (AL150) for 8 weeks during natural heat stress

In assessing hepatic redox status, all feed additive groups notably decreased MDA levels (*P* < 0.05). Supplementing with 150 mg of AL notably increased TAC in hepatic tissues compared to the heat-stressed group. Stressed growing rabbits fed diets with 40 mg of VE, 5 mg of VC, and 150 mg of LYC showed similar results for MDA compared to the control group (*P* > 0.05). All dietary supplements, except VE40, significantly decreased liver tissue MDA levels compared to the heat-stressed group (*P* < 0.05) (Table 6).

### Immunity and pro-inflammatory cytokines

The levels of Immunoglobulin G in the treated groups were significantly higher than in the HS treatment group (*P* < 0.05), while the levels of Immunoglobulin A did not differ among all experimental groups (*P* > 0.05; Table 7). Stressed rabbits in the AL150 group had the lowest levels of TNF-α compared to the HS group (*P* < 0.05). There were no statistically significant differences among all supplemented groups for TNF-α (*P* > 0.05). The levels of IL-4 were significantly decreased by the addition of various antioxidants except for the VE40 group (*P* < 0.05). All feed additives significantly decreased the levels of IFN-γ in the serum of stressed growing rabbits compared to the HS group (*P* < 0.05). The supplementation of antioxidants to the diets of stressed rabbits had no significant effects on the values of lysozyme activity (*P* > 0.05).

**Table 7:** Effect of dietary different sources of antioxidants supplementation on immunoglobins and inflammatory response of fattening rabbits exposed to heat stress conditions

Variables	Experimental groups^1^					SEM	*P*-value
	CON	VE40	VC5	LYC150	AL150		
**Immunoglobulins**							
IgG (ng/mL)	47.89^b^	50.86^ab^	55.4^a^	55.32^a^	55.17^a^	2.98	0.001
IgA (ng/mL)	19.25	20.93	23.34	23.44	23.35	1.96	0.004
**Inflammatory cytokines**							
TNF-α (pg/mL)	87.38^a^	82.15^ab^	77.64^ab^	77.43^ab^	72.73^b^	3.96	0.0096
IL-4 (pg/mL)	116.11^a^	108.96^ab^	98.99^b^	101.09^b^	96.27^b^	4.43	0.0294
IFN-γ (pg/mL)	85.54^a^	78.71^b^	72.87^c^	76.92^b^	75.47^b^	1.04	0.020
Lysozyme (pg/mL)	2.09	2.23	2.27	2.29	2.34	0.31	0.102

^ac^ Means not sharing a common superscript in a row are significantly different (*P* < 0.05). Immunoglobulin G (IgG), G (IgM) tumor necrosis factor-alpha (TNF-α), interferon-gamma (IFN-γ), and interleukin-4 (IL-4). ^1^ Growing rabbit fed basal diet without feed additive (CON group and serve as HS group) or fed diets with 40 mg of vitamin E (VE40), 5 mg of vitamin C (VC5), 150 mg of lycopene (LYC150) or 150 mg/kg diet of allicin (AL150) for 8 weeks during natural heat stress

## Discussion

This study concludes that different natural antioxidant sources can help to mitigate the adverse effects of HS in rabbits, as evidenced by improvements in growth, immunity, and overall health of fattening rabbits. Allicin (AL) has been shown to be the most effective natural molecule in enhancing blood health, growth, feed utilization, redox status, and reducing proinflammatory cytokines in stressed growing rabbits. The adverse consequences of HS in growing rabbits can be alleviated through various approaches, including nanoparticles, essential oils, and vitamins. In our study, the recorded THI value indicated that the growing rabbits were exposed to severe HS (THI values was 29.76 ± 0.20 during the experimental period). THI was effectively used to determine the severity of HS in various animals including rabbits.

This study shows that the addition of various natural substances (AL or LYC) or vitamins (E or C) significantly improved the FBW in growing rabbits exposed to HS when compared to the HS group. HS weakened the LBW in growing rabbits as noticed by several scientists [7, 20, 36]. However, the addition of LYC (200 mg) or AL (200 mg) in rabbit diets significantly improved the FBW in rabbits and had no significant effects on FI [20]. Additionally, a study by [10] indicated that the supplementation of tomato powder (high content of LYC) improved the overall growth performance of growing rabbits. In contrast to our findings, a study by [25], reported that the addition of AL or LYC had no substantial effect on FI and LBW in growing rabbits, but FCR was improved by the addition of LYC and AL. Additionally, vitamins (E and C) significantly improved the growth indices of stressed rabbits [9, 15]. The enhanced growth indices observed in response to dietary natural compounds during heat stress might be attributed to their protective effects on the body and potential stimulation of digestive enzymes [10]. However, the direct impact on digestive enzymes was not measured in this study.

Mitigating the adverse effects of HS with natural compounds may be attributed to the antioxidants’ abilities to scavenge the OS induced by HS in the cellular system. The positive influences of AL on growth performance have been demonstrated in studies [20]. It was reported that AL stimulates intestinal flora, improves digestion, increases cecum anaerobic bacteria count [7], and inhibits the growth of bacteria (pathogenic and non-pathogenic types) in the gastrointestinal tract, consequently improving the growth efficiency of rabbits [37]. Additionally, garlic supplementation in broiler diets at a concentration of 200 mg/kg enhances pancreatic enzyme activity and promotes better nutrient absorption during HS [6]. Vitamins (C and E) have been shown to significantly enhanced the growth and feed intakes in growing rabbits [9].

Dietary interventions to enhance functional meat and reduce the negative effects of HS in animals’ diets are interesting areas to explore. In this study, the supplementation of various natural substances did not affect the meat quality, except dressing percentage. The group given AL had a significantly higher dressing percentage than the other treatments and HS group. Similar conclusions were reported by [20, 25]. In contrast, it has been clarified that the addition of LYC improves the nutritive value of rabbit meat in relation to human health [38].

The hematology profile provides valuable insights into the overall health of animals. Supplementation with various natural compounds did not alter the blood health, except for hemoglobin (Hb) levels. Rabbits fed diets containing natural compounds showed higher Hb levels than the HS group, indicating that these molecules can prevent anemia induced by HS in rabbits. The addition of LYC or AL had no significant effects on the hematology profile in rabbits [25]. This is consistent with a study [10], that found that tomato powder (2%) had no effects on haematological parameters (except for RBCs and WBCs), while [39] reported that the addition of garlic extracts significantly improved all the hematology indices of rabbits. Vitamin C acts as an antioxidant, reducing hypercholesterolemia and protecting against damage due to OS in heat-stressed rabbits [12]. Generally, natural antioxidants may have contributed to the improvement in blood physical characteristics like RBCs and WBCs, as these nutrients are essential for cell formation and hemoglobin production. Similar results were stated by other scientists [9, 15]. Vitamin E has antioxidant action due to its robust scavenging capabilities [40] [12]. reviewed the potential benefits of vitamin C in enhancing heat resistance in growing rabbits. AL is the main component of garlic oils. Previous studies have found that garlic oil supplementation in stressed broiler diets significantly decreased liver enzymes (AST and ALT), kidney function (creatinine and uric acid), and lipid profile [6], , which is consistent with our data.

Additionally, activity of AST, and ALT, as well as the concentrations of creatinine and urea were significantly reduced in the serum of growing rabbits fed LYC (5 mg /kg diet) [40]. These results confirm the hepatoprotective, hypocholesterolemic and hypoglycemic of LYC [41]. In mice with fatty liver disease, AL attenuates the oxidative stress and inflammation by reducing fat accumulation, liver enzymes and the expression of microsomal protein cytochrome P450 2E1 (CYP2E1) [42]. Furthermore, rabbit fed wit 10 mg of LYC had lower levels of lipid profiles than the control group. In rabbits fed a high-fat diet, LYC was observed to considerably reduce lipid parameters [19]. The same data was also shown in rabbit bucks fed diets with LYC [16]. Studies have shown that dietary vitamin C improves growth performance, increases antioxidant enzyme levels, and reduces triglycerides and oxidative stress markers in heat-stressed rabbits [5, 17].

It is well known that HS can trigger inflammation, apoptosis and oxidative stress in the hepatic tissues of growing rabbits. The enhancement of protein fractions in all supplemented groups demonstrates the ability of these feed additives to enhance the secretion of intestinal enzymes, thereby improving absorption and protein synthesis in liver [8, 43]. Similar to our data, a study by [20] found that LYC and AL can improve liver function during adverse environmental impacts. AL can enhance intestinal function by improving the beneficial bacteria in the intestines, especially those related to protein digestion.

Antioxidant defense system is crucial for maintaining normal physiological pathways in the cellular system. Elevated temperature triggers a redox imbalance by increasing MDA levels and decreasing GSH, SOD, and TAC. Our results suggest that antioxidant supplementation can effectively raise the levels of antioxidant biomarkers in both serum and hepatic tissues, indicating a potential protective effect against oxidative damage. The antioxidant capacity of LYC and AL was confirmed in rabbit bucks, as reported by [16]. This finding highlights the importance of incorporating antioxidants into the diet during challenging conditions to support overall health and well-being. Like our results, many studies have indicated that garlic oil (a main source of AL) can improve GSH and CAT as antioxidant enzymes and significantly decreased MDA levels in broilers [6]. In addition, AL reduced total cholesterol, triglycerides, platelet aggregation, ocular pressure, alcohol dehydrogenase and abundant cysteine proteinase activities [23]. Moreover, the antioxidant, antimicrobial and anti-inflammatory of AL have been confirmed [42]. Allicin has been found to have an anti-inflammatory pathway and neuroprotective impact against brain ischemia, leading to significant reductions in the levels of TNF-α and MDA [44]. Furthermore, a study involving stressed rabbits fed with AL (100 mg/kg diet) showed control of TNF-α gene expression in the liver, which alignment with our results [20].

HS induces immune dysfunction in animals, leading to inflammation and apoptosis processes. Therefore, adding a feed additive to support immunity and reduce inflammation may be crucial for optimizing productivity during climate changes [36, 45]. In this study, we observed that all supplemented groups showed improvements in IgG, while having no significant effects on IgA. IgG is the most abundant type of antibody in serum, accounting for 75% of total antibodies. It is a glycoprotein produced by the immune system to combat foreign substances and microbes [46]. Allicin inhibits xanthine oxidase, an enzyme that produces superoxides, by interacting with the enzyme’s thiol groups [24]. Allicin has antioxidant properties that can help to protect cells from damage caused by OS. This inhibition reduces ROS production, helping to alleviate oxidative stress [11]. AL may also act as a precursor to biological agents or regulate thiol proteins, ultimately reducing OS in cells.

Inflammatory cytokines including TNF-α, IL-4, and IFN-γ were significantly decreased by HS in rabbits as reported by [7, 16, 36]. Recently, targeting inflammation may enhance the health and productivity of animals and help them cope with heat stress. LYC has been shown to have anti-inflammatory action by decreasing the expression of TNF-α in hepatic tissue of growing rabbits [20]. Therefore, LYC could be a suitable treatment for managing palmitic acid-induced cardiotoxicity in female rats due to its robust antioxidant status and reducing inflammation actions [18]. A study by [22] found that LY has an anti-inflammatory effect [22] via inhibiting the expression of NF-κB expression and serum levels of IL-4 in rats. Moreover, the anti-inflammatory effects of LYC, observed in numerous studies, primarily stem from its ability to modulate pathways involved in the induction of inflammatory mediators and the NF-κB signaling pathway. Lycopene exerts its anti-inflammatory action by binding to the IκB protein, preventing its dissociation and subsequent translocation of NF-κB from the cytoplasm to the nucleus [20]. This data was confirmed by a histological study on hepatic tissues as reported by [20], who found that LYC or AL had hepatoprotective effects in growing rabbits. IFN-γ is critical for innate and adaptive immunity against viruses and bacteria, whereas higher levels of IFN-γ during HS conditions in rabbits [7, 36], induce higher inflammatory cells. All feed additives significantly decreased the levels of IL-4 and IFN-γ in rabbit serum exposed to HS [11]. AL or LYC act as anti-inflammatory agent that limit the effects of pro-inflammatory cytokines. AL acts as an anti-inflammatory agent through inhibition of NF-κB activation, and inhibition of NLRP3 inflammasome activation, thereby reducing the production of inflammatory cytokines [11]. Lysosome activity did not differ among all groups. In contrast with our results [11], found that thymoquinone significantly improved lysosome activity during HS in rabbits. One limitation of this study is the lack of molecular mechanistic understanding to determine the best anti-stress agent. Future research will focus on metabolic changes induced by heat stress and explore additional pathways to enhance our understanding of the effects of environmental changes on animals.

## Conclusion

This study examines the use of natural interventions to alleviate the adverse effects of heat stress (HS) in growing rabbits. A comparative study indicates that lycopene (LYC, 150 mg/g diet) or allicin (AL, 150 mg/kg diet) can protect against heat stress-induced growth retardation, blood alterations, immune dysfunction, and inflammation in growing rabbits. These beneficial effects may be due to the antioxidant and anti-inflammatory properties of these compounds compared to vitamins E or C. Additional research is necessary to confirm these findings through proteomic and molecular analyses in rabbits subjected to hot environments and supplemented with natural dietary interventions.

## Data Availability

All data included in this study are presented in the form of tables and figures.
